# Use of statins and the risk of delirium in critically ill and surgical patients: Protocol of a systematic review and meta-analysis

**DOI:** 10.1097/MD.0000000000013679

**Published:** 2018-12-21

**Authors:** Hai Zeng, Zunjiang Li, Guoxin He, Yanhong Han, Wenbin Fu, Junru Wen

**Affiliations:** a The Second Clinical College; b The First Clinical College, Guangzhou University of Chinese Medicine, Guangzhou; c Department of Acupuncture and Moxibustion, Guangdong Provincial Hospital of Chinese Medicine; d Shanghai University of Chinese Medicine; e Shanghai University of Medicine & Health Sciences, Shanghai, China.

**Keywords:** delirium, meta-analysis, protocol, statins, systematic review

## Abstract

**Background:**

The critically ill and surgical patients are at significant risk of delirium, which is associated with a high morbidity and mortality. The association between statin use and the incidence of delirium is still controversial. In this article, we will perform a systematic review and meta-analysis of published studies to evaluate the effectiveness of statins for the prophylaxis of delirium among critically ill and surgical patients.

**Methods:**

We will conduct a systematic literature search in EMBASE, PubMed, and the Cochrane Library from inception date to October 2018 for randomized controlled trials (RCTs) and observational studies (either cohort or case-control studies) investigating the association between use of statins and delirium risk. The Cochrane Collaboration's tool for evaluating the risk of bias and Newcastle-Ottawa scale (NOS) will be used to assess the methodological quality of RCTs and observational studies, separately. The primary outcome will be the risk of incident delirium associated with statin use. Pooled odds ratios (ORs) with corresponding 95% confidence intervals (CIs) will be calculated by a random-effects or fixed-effects model according to heterogeneity among included studies. Subgroup analyses, meta-regression method, and assessment of publication bias will be also performed. Statistical analyses will be conducted with RevMan (version 5.3.5) and Stata (version 14.0) software. In addition, the grading of recommendations assessment, development and evaluation (GRADE) approach will be applied to evaluate the quality of evidence.

**Results:**

The study will provide a high-quality synthesis and evaluate the effectiveness of statins for delirium prevention among critically ill and surgical patients.

**Conclusions:**

The systematic review and meta-analysis will provide convincing evidence concerning the effect of statins against delirium in critically ill and surgical patients.

## 1 Introduction

Delirium is a syndrome of acute brain dysfunction, its occurrence is associated with increased risk of mortality and morbidity, length of hospital stay, institutionalization, long-term cognitive impairment, permanent dementia, and burden of health care costs.^[1–4]^ A recent systematic review has reported that delirium occurred in 11% to 51% of surgical patients and 19% to 82% of critically ill patients, moreover, there is greater risk in elderly patients aged 65 years and older.^[1]^ Around one-third of delirium cases are considered to be potentially preventable, and to modify key clinical factors which may precipitate delirium, a multicomponent intervention package is recommended for the patient population at high risk, including orientation communication, non-pharmacologic approaches to promote adequate duration and quality of sleep, hypoxaemia prevention, infection control, and pain management.^[5]^ Whereas no pharmaceutical monotherapy has been proved to decrease the incidence of delirium in adult patients based on convincing evidence.^[1,6–8]^

Despite the importance of this neuropsychiatric syndrome, its pathophysiology remains poorly understood. Recently, the inflammatory alteration in the central nervous system has been recognized as an important factor involved in delirium pathophysiology.^[9,10]^ Statins, as inhibitors of the 3-hydroxy-3-methylglutaryl coenzyme A (HMG-CoA) reductase to treat hypercholesterolemia, have been applied to prevent major cardiovascular events.^[11]^ Further, statins exert pleiotropic properties, including anti-inflammatory, immunomodulatory, and antithrombotic effects.^[11–13]^ These pleiotropic effects may contribute to prevent or mitigate delirium in critically ill and surgical patients by modifying the process of neuroinflammation and activation of proinflammatory microglia.^[8,10,14–17]^

Over recent years, an increasing number of studies have investigated the efficacy of statins on the prophylaxis of delirium in critically ill and surgical patients. A previous meta-analysis^[18]^ suggested that statins might not decrease the occurrence of delirium in critically ill and cardiac surgery patients. However, the review only included 6 observational studies that published before or during 2014. Recent randomized controlled trials (RCTs) and observational studies have yielded conflicting conclusions about the prophylactic efficacy of statins on delirium, therefore, there still remains uncertainty concerning the topic. As current studies continue to provide new evidence, we plan to conduct this review to reappraise available evidence regarding the effectiveness of statins on delirium among critically ill and surgical patients.

## 2 Methods

The meta-analysis will be performed in accordance with the preferred reporting items for systematic review and meta-analysis protocols (PRISMA-P) statement^[19,20]^ and preferred reporting items for systematic reviews and meta-analyses (PRISMA) criteria.^[21]^ The protocol of our review has been registered in the international prospective register of systematic reviews (PROSPERO) (registration no. CRD42018111324).

### 2.1 Inclusion and exclusion criteria for study selection

#### 2.1.1 Types of studies

Included studies will be required to investigate statin use and delirium risk. The study designs will contain RCTs and observational studies (either cohort or case-control studies).

#### 2.1.2 Types of participants

The population of interest will include adult patients (aged 18 years or older) admitted to intensive care unit (ICU) or undergoing general anesthesia surgery (either cardiac or non-cardiac surgery) regardless of race, region, sex, and age.

#### 2.1.3 Types of interventions and controls

For included RCTs, the intervention received by patients will be any type of statins, including atorvastatin, cerivastatin, fluvastatin, pitavastatin, pravastatin, rosuvastatin, simvastatin, and other statins, while a placebo will be administrated in the control group. For included observational studies, it will be required to investigate the effects of statin therapy compared with non-statin use, and drug exposure of participants will be the use of any statin.

#### 2.1.4 Types of outcomes

The outcomes of interest will be the risk of incident delirium associated with statin use. The assessment methods of delirium need be reported, for example, the Confusion Assessment Method (CAM), CAM for ICU, Intensive Care Delirium Screening Checklist (ICDSC). There will be sufficient published data for obtaining or calculating the estimates of crude or adjusted odds ratios (ORs) with corresponding 95% confidence intervals (CIs) of delirium risk related to use of statins in eligible studies. For each observational study, we will consider the estimate which is the most fully controlled for potential confounders, when 2 or more estimates of effects (ORs) are presented.

#### 2.1.5 Exclusion criteria

Case reports, trials that did not report clinical outcomes of interest, review articles, comments, letters, conference abstracts, animal experiments, in vitro experiments, and duplicated reports will be eliminated.

### 2.2 Search strategy

Two independent reviewers will conduct a systematic literature search. Reviewers will search for articles evaluating statin use and delirium risk in several databases, including PubMed (from 1946 to October 2018), EMBASE (from 1974 to October 2018), and the Cochrane Library database (from 1974 to October 2018). The publication language will be restricted to English. The study designs will include RCTs, retrospective or prospective cohort and case-control studies. The Medical Subject Headings (MeSH) or non-MeSH terms and their combinations will be applied to search in the title and abstract. The complete search strategy in PubMed is provided as an instance in supplemental material. Any disagreement will be resolved by discussing among reviewers. Meanwhile, we will also search for recent meta-analyses and review articles regarding the topic for reviewing references to supplement additional related studies.

### 2.3 Data collection

#### 2.3.1 Selection of studies

Articles identified by the initial computerized literature search will be imported into the document management system of EndNote (version X8, Thomson ResearchSoft, Connecticut, USA) software, which could manage literature effectively and remove duplicate records automatically. Two reviewers will eliminate unqualified articles independently by screening the titles and abstracts based on the pre-specified inclusion and exclusion criteria. Further, the surplus articles will be scrutinized via reviewing in full text. Finally, remaining eligible studies that meet inclusion criteria will be included in our review. Different opinions between reviewers will be solved by a discussion with another reviewer. The process of studies selection will be depicted in PRISMA flowchart.

#### 2.3.2 Data extraction

Any eligible study need meet all the requirements of inclusion criteria. Two independent reviewers will extract data from included studies using a standard data abstraction sheet. The reviewers will get to consensus by discussions to settle all disagreements. Data abstracted will incorporate the first author's name and published year, publication journal, basic characteristics of participants (average age, sex ratio, and total sample size), countries where studies were performed, patient types, statins used in included studies (types, doses, and durations), definition of statin exposure and control for confounding factors by adjustments or matching in observational studies, assessment methods of delirium, the outcomes of interest mentioned above, the duration of the follow-up.

#### 2.3.3 Dealing with missing data

If there exist missing data in the included literature, 2 reviewers will send e-mails to contact the first or corresponding author to acquire missing information. If sufficient data could not be obtained in this way, available data will be used for data synthesis. Additionally, the possible influence of the missing data on the results of our review will be discussed.

### 2.4 Quality assessment of included studies

Using the Cochrane Collaboration's tool for evaluating the risk of bias, 2 independent reviewers will assess the qualities of included RCTs. The tool contains 7 quality items: random sequence generation, allocation concealment, blinding of participants and personnel, blinding of outcome assessment, incomplete outcome data, selective reporting, and other bias.^[22]^ Of those, each item will be assigned a judgment of low, unclear, or high risk of bias. The Newcastle-Ottawa scale (NOS)^[23]^ will be applied to assess the quality of cohort and case-control studies by 2 independent reviewers, including 3 dimensions as follows: patient selection, comparability of cases and controls (for case-control studies) or exposed cohorts and non-exposed cohorts (for cohort studies), and ascertainment of exposure (for case-control studies) or assessment of outcome (for cohort studies). The scores of NOS range from 0 up to 9 points. Studies with a score of 6 or more points will be judged to be of high quality while remaining studies will be considered to be of low quality. We will resolve all discrepancies by discussions among reviewers.

### 2.5 Statistical analysis

#### 2.5.1 Data synthesis and analysis

For the meta-analysis, the effect size (ES) with 95% CI will be defined as ORs and 95%CI to indicate the difference of the incident rate of delirium as a function of statin therapy. Notably, the differences in the estimated magnitude of effects between RCTs and observational studies are common, thus, 2 separate meta-analyses will be performed, which will inspect consistency of results across different study designs. Statistical analyses will be performed with RevMan (version 5.3.5, the Nordic Cochrane Centre, Copenhagen, Denmark) and Stata (version 14.0, StataCorp, College Station, TX, USA) software. All analyses will be undertaken at the .05 level of significance. The *I*^*2*^ statistic will be used to calculate heterogeneity among included studies quantitatively. An *I*^*2*^ statistic is deemed to represent no (0%), low (0%–25%), moderate (25%–75%), and high (75%–100%) potentiality of heterogeneity, respectively.^[24]^ If there exists moderate or high heterogeneity among included studies, a random-effects model will be applied to calculate the aggregated estimate of effects, otherwise, the data will be synthesized by a fixed-effects model.

#### 2.5.2 Subgroup and meta-regression analyses

We will perform subgroup analyses according to study designs (cohort and case-control studies), countries where studies were performed, patient types (patients admitted to medical ICU, cardiac or non-cardiac surgery patients), and types of statins involved in included studies. In addition, when substantial heterogeneity is observed among included studies, we will also conduct a meta-regression analysis of these variables to explore potential sources of heterogeneity.

#### 2.5.3 Sensitivity analysis

To identify the stability of the review conclusions, we will conduct sensitivity analysis based on sample size and the methodological quality of included studies.

#### 2.5.4 Assessment of publication bias

The Egger regression test and the Begg rank correlation will be performed to assess publication bias upon visual measurement of the funnel plot if there is an adequate number of included studies (10 or more studies).

### 2.6 Grading the quality of evidence

The grading of recommendations assessment, development and evaluation (GRADE)^[25]^ approach will be applied to evaluate the quality of evidence for the effects of statins on delirium prevention. The quality of evidence includes four grades as follows: high, moderate, low, and very low.

### 2.7 Ethics and dissemination

The study is a meta-analytic review. All data included in this study will be abstracted from published articles and not involve personal privacy of patients, thus, ethical approval is not required. The results of our review will be published in a peer-reviewed publication.

## 3 Discussion

Delirium is an acute confusional state, whose clinical features involve fluctuating course, inattention, disorganized thinking, altered level of consciousness, cognitive deficits, perceptual disturbances, psychomotor disturbances, altered sleep-wake cycle, and emotional disturbances.^[8]^ The older patients are the high-risk population of delirium, especially those who are admitted to ICU or undergoing general anesthesia surgery.^[1,8]^ The exact mechanisms that contribute to the development of delirium are not elucidated clearly. There is a hypothesis that the neurotransmission, inflammation, and chronic stress are involved in the pathogenesis of delirium.^[1,2,8,26]^ Of those, neuroinflammation is an important contributing factor to the occurrence of delirium according to published work.^[9,10]^ The inflammatory response in the human brain may be stimulated by peripheral inflammation.^[27,28]^

Known as lipid-lowering agents, statins are recommended in cholesterol guidelines for primary and secondary prevention of atherosclerotic cardiovascular disease (ASCVD).^[29]^ Among patients undergoing non-cardiac surgery, perioperative statin treatment also improves cardiac complications.^[30]^ In a systematic review,^[31]^ pooling of results from 25 prospective cohort studies have revealed that statins might exert cognitive protective effects on mild cognitive impairment, all-cause dementia, and Alzheimer disease. More importantly, in recent years, several studies have suggested that the use of statins was associated with a reduced risk of delirium, however, some studies tend to reveal no considerable preventive effect. To date, the potential mechanisms of benefit of statin therapy to decreased delirium incidents are poorly understood and likely associated with its pleiotropic properties. Thus, we intend to conduct a systematic review and meta-analysis of published studies to provide the most comprehensive evidence concerning the effects of statins on incident delirium among the critically ill and surgical patients.

Additionally, a prior review^[18]^ observed no favorable effect concerning statins for delirium prevention which conducted a combined meta-analysis of only 6 studies undertaken in critically ill and cardiac surgery patients. Nonetheless, compared to the previous study, RCTs and more observational studies will be enrolled in the present meta-analytic review, resulting in a larger dataset for data synthesis and analysis. Moreover, the prior study was lack of necessary analyses with respect to different patient populations. Given the clinical heterogeneity among medical critical illness, non-cardiac, and non-cardiac surgery, we plan to analyze these subgroups respectively. Additionally, the published review regarded all statins together as a class effect, thus, there is still a paucity of meta-analysis concerning different statins. Importantly, recent studies observed inconsistent results concerning the protective effect of lipophilic (eg, simvastatin and atorvastatin) and hydrophilic (eg, pravastatin and rosuvastatin) statins on delirium. Therefore, we can not exclude differential effects of a particular statin on delirium in the previous review, subgroup analyses stratified by different types of statins will also be performed in our meta-analytic review.

Nevertheless, several limitations in our review should be addressed. The different diagnostic criteria for delirium among included studies might influence the comparability and integration of enrolled researches in the review. In addition, only English literature will be included in our review, thus studies published in other languages may be missed.

## Author contributions

**Conceptualization:** Hai Zeng, Junru Wen, Yanhong Han.

**Data curation:** Guoxin He, Zunjiang Li, Junru Wen, Yanhong Han.

**Formal analysis:** Hai Zeng, Zunjiang Li, Yanhong Han, Junru Wen.

**Funding acquisition:** Hai Zeng, Junru Wen, Wenbin Fu.

**Investigation:** Hai Zeng, Zunjiang Li, Junru Wen.

**Methodology:** Hai Zeng, Yanhong Han, Junru Wen,Wenbin Fu.

**Project administration:** Guoxin He.

**Resources:** Hai Zeng, Guoxin He.

**Software:** Hai Zeng, Zunjiang Li,Yanhong Han, Junru Wen, Wenbin Fu.

**Supervision:** Hai Zeng, Junru Wen, Wenbin Fu.

**Visualization:** Hai Zeng, Zunjiang Li.

**Writing – original draft:** Zunjiang Li, Guoxin He, Junru Wen.

**Writing – review & editing:** Hai Zeng, Yanhong Han, Wenbin Fu, Junru Wen.
